# Narrow-band random Raman lasing from Rhodamine 6G assisted by cascaded stimulated Raman scattering effect

**DOI:** 10.1038/s41598-021-01354-8

**Published:** 2021-11-05

**Authors:** Mandana Sadat Hosseini, Elnaz Yazdani, Batool Sajad

**Affiliations:** 1 Department of Physics, Tarbiat Modares University, P.O. Box 14115-175, Tehran, Iran; 2 Department of Physics, Faculty of Physics and Chemistry, Alzahra University, Tehran, Iran

**Keywords:** Optical materials and structures, Nanocavities, Optics and photonics, Lasers, LEDs and light sources

## Abstract

This study reports the first experimental observation of cascaded stimulated Raman scattering (SRS) generation in a colloidal disordered medium. Generation of the cascaded effect requires both a high Raman gain and pump power in the disordered medium. Here, to extend effective path lengths of photons into the Raman gain medium for producing additional SRS processes, ZnO microspheres with abundant nano-protrusions as suitable scattering centers are proposed. It is explained that nano-protrusions on the surface of the spheres can act as nano reflectors and significantly provide potent feedback in the disordered system. This provided feedback via nano-protrusions boosts cascaded SRS generation to allow the appearance of higher Raman signals of Rhodamine 6G dye solution at a low scatterer concentration of 5 mg/ml. The threshold for the formation of the first Raman signal is measured at about 60 mJ/pulse. Also, the evolution of Raman signals under several fixed pump pulses is examined to investigate the stability from pulse to pulse. Our findings provide promising perspectives for achieving the single-frequency laser sources and generate desirable wavelengths for specific applications.

## Introduction

Stimulated Raman scattering (SRS) is a nonlinear optical process that occur when the energy from a high power pump beam transfer to a probe beam and the frequency difference between the pump and the scattered stokes photons matches a given molecular vibrational frequency of the medium^1^. The SRS effect shows itself in the form of a gain of the Stokes beam power (stimulated Raman gain, SRG) and a loss of the pump beam power (stimulated Raman loss, SRL)^1,2^. On the other hand, when the intensity of the generated Stokes signal becomes sufficiently high, the cascaded SRS effect can appear^3^. The term cascade SRS effect refers to the fact that this intense signal can act as the pump for a further Raman process^3–5^. High Raman gain of the material and high pump power is required to achieve the effect^6–8^. Finally, this cascaded process can continue and generate higher Stokes lines at longer wavelengths.

Raman lasers are specific lasers whose light amplification mechanism is fulfilled by the SRS process^9^. In recent years, one kind of Raman lasers has been introduced as the random Raman laser and have attracted extensive attention due to their simple cavity-less configuration and wavelength stability^5,10–12^. Random Raman lasers operate on a similar gain mechanism (i.e., SRS process) as Raman lasers, while multiple elastic scattering provides the feedback mechanism instead of a cavity^13,14^. Generally, the optical sources of disordered gain media in which the multiple elastic scattering of light provides feedback in place of a resonator are known as random lasers^15–17^.

To date, the competition between SRS and random lasing in disordered media has been observed in various systems^11,12,18–25^. Colloidal solutions and metamaterials have also been offered for tuning elastic scattering to enhance the Raman signals generation^26^. But, observation of several Stokes orders (cascaded SRS generation) is only reported in systems such as fibers^4–7,27–30^, Raman crystal under high-intensity pump pulses^31–34^, and the barium sulfate (BaSO_4_) powders^18^, in our knowledge.

In this study, the evolution of the SRS signal was measured as a function of the laser pump energy in Rhodamine 6G as a Raman gain medium. To the best of our knowledge, the cascaded SRS generation in a colloidal Raman gain medium is observed under the high pump intensity for the first time. Here, Zinc Oxide was chosen as the scatterer center due to its low absorption at the pump excitation wavelength (i.e. 532 nm) and its high refractive index (n ≃ 2) to provide strong scattering in the random medium. This particular morphology of ZnO microspheres as the scatterer centers, with the nano-porous surface, was synthesized and used to enhance feedback mechanism and Raman gain for boosting cascaded SRS generation. Observation the higher Raman signals in Rhodamine 6G solution could be attributed to the increased multiple elastic scattering and the additional SRS process via this particular morphology, which helps to cascaded SRS generation. The strong multiple elastic scattering could be satisfied by the porous structure of ZnO microspheres, and nano-protrusions on the surface of each sphere act as nano reflectors to provide efficient feedback. Therefore, by adding ZnO microspheres to Rhodamine 6G solution, the generation of Raman signals at a low threshold in a simple setup has been achieved compared to the previous study in Ref.^35^. Moreover, the evolution of the Raman signals under some fixed pump energies has been evaluated to study their stability.

In addition to a low-cost material and simple experimental setup, this approach opens the path to achieving the single-frequency laser sources with many applications which isn't easy to generate desirable wavelengths for specific applications.

## Experimental materials and setup

### Synthesis of ZnO microspheres

ZnO microspheres were prepared by the hydrothermal synthesis approach. First, 0.004 mol of zinc acetate dihydrate (Zn(CH_3_COO)_2_–2H_2_O) was dissolved in 40 ml of diethylene glycol. The solution was then transferred into a Teflon-lined autoclave (75 mL). The autoclave was sealed and placed into an oven. The temperature was increased from room temperature to 160 °C in 20 min and was maintained for 5 h. After completing the reaction time, the autoclave was naturally cooled down to room temperature. The white products were cleaned by three centrifugation/washing/re-dispersion cycles in ethanol and dried on a heater at 120 °C^36^.

### Characterization

The morphology and structure of the ZnO microspheres were determined using field-emission scanning electron microscopy (FESEM, MIRA3 TESCAN). In addition, the Raman spectrum of Rhodamine 6G dye was measured by Raman spectrometer (Tekram, P50C0R10) with an exciting laser wavelength of 532 nm and power of 10 mW in the range 200–1900 cm^−1^.

### Experimental details

Rhodamine 6G (C_28_H_31_N_2_O_3_Cl), with a molecular weight of 479.02 g/mol and methanol (CH_3_OH) with spectroscopic grade purity, was supplied by Sigma-Aldrich and Merck Chemical companies, respectively.

For the sample preparation, the concentration of Rhodamine 6G in methanol as a gain medium was fixed at 5 × 10^−3^ M. Then, ZnO microspheres as scatterer centers with a 5 mg/ml concentration were added to the Rhodamine 6G solution. To avoid the microspheres sedimentation, the solution, contained in a flask, is shaken in an ultrasonic cleaner for 15 min right at the beginning of the experiment.

**Figure 1:**
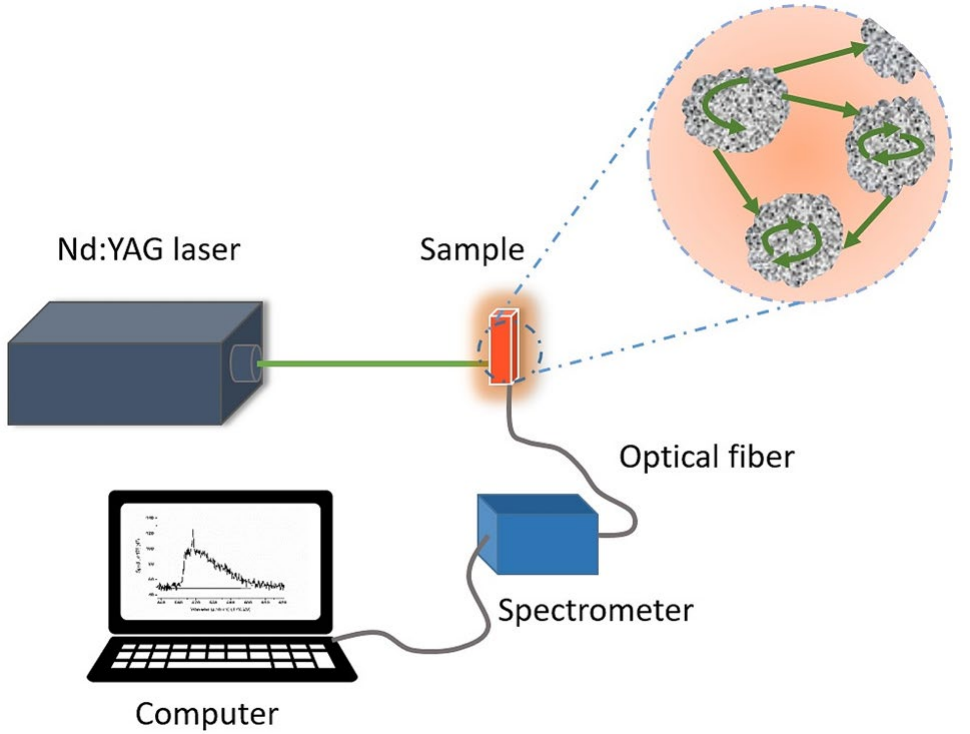
Schematic experimental setup of the random Raman laser experiment.

A quartz cuvette with four transparent windows (12.5 × 12.5 × 40 mm) containing 2 mL of the solutions was used as a container in the experimental setup. The sample is pumped using a laser pulse of 532 nm from a Q-switched Nd:YAG laser model Brilliant made by Quantel with a pulse duration of 10 ns. The diameter of the pumping laser spot was about 3 mm, and the pumping laser was at normal incidence to the sample. A fiber bundle collected all emission spectra, UV 600 (core)/660 (cladding) type with SMA-905 at an angle of 90 degrees to the optical axis set by the excitation light beam. The fiber output was coupled to the entrance slit of a compact wide range spectrometer (200–1100), model S150 Solar Laser System TM, with a spectral resolution of 0.4 nm. A charge-coupled device array detector, model Toshiba TCD 130AP was used to detect the emission signals. All measurements have been performed at room temperature. The experimental setup is schematically shown in Fig. 1.

## Results and discussion

**Figure 2:**
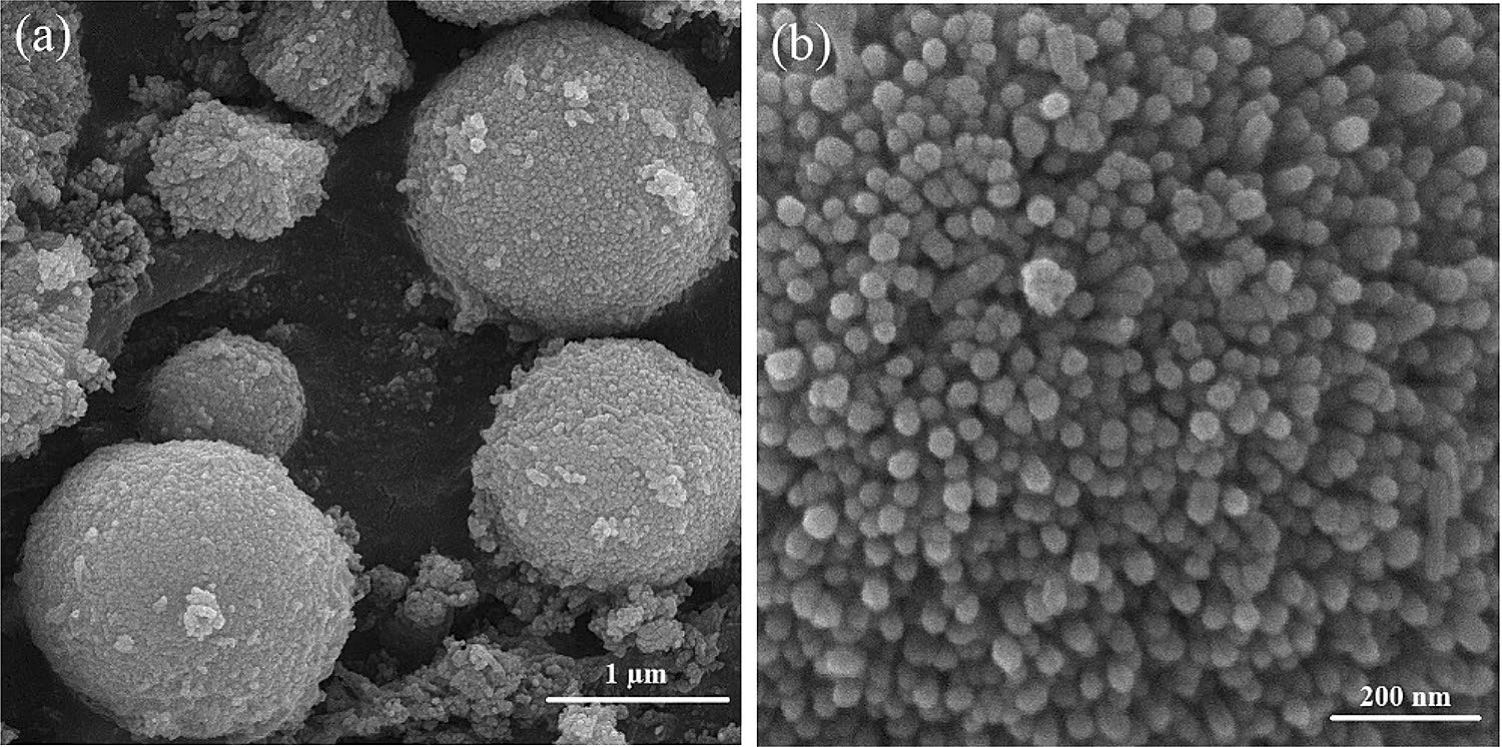
Field emission scanning electron microscopy (FESEM) images of the ZnO microspheres: (**a**) at scale bar 1 μm and (**b**) 200 nm from the surface of a microsphere.

Figure 2a represents the FESEM image of the ZnO microspheres to show the microspheres with rough surfaces and average diameters of about 1 μm. Further magnification from the microsphere’s surface in Fig. 2b signifies that the individual microsphere comprises nano-protrusions with average diameters of ~ 30 nm, which are uniformly grown very closely spaced.

**Figure 3:**
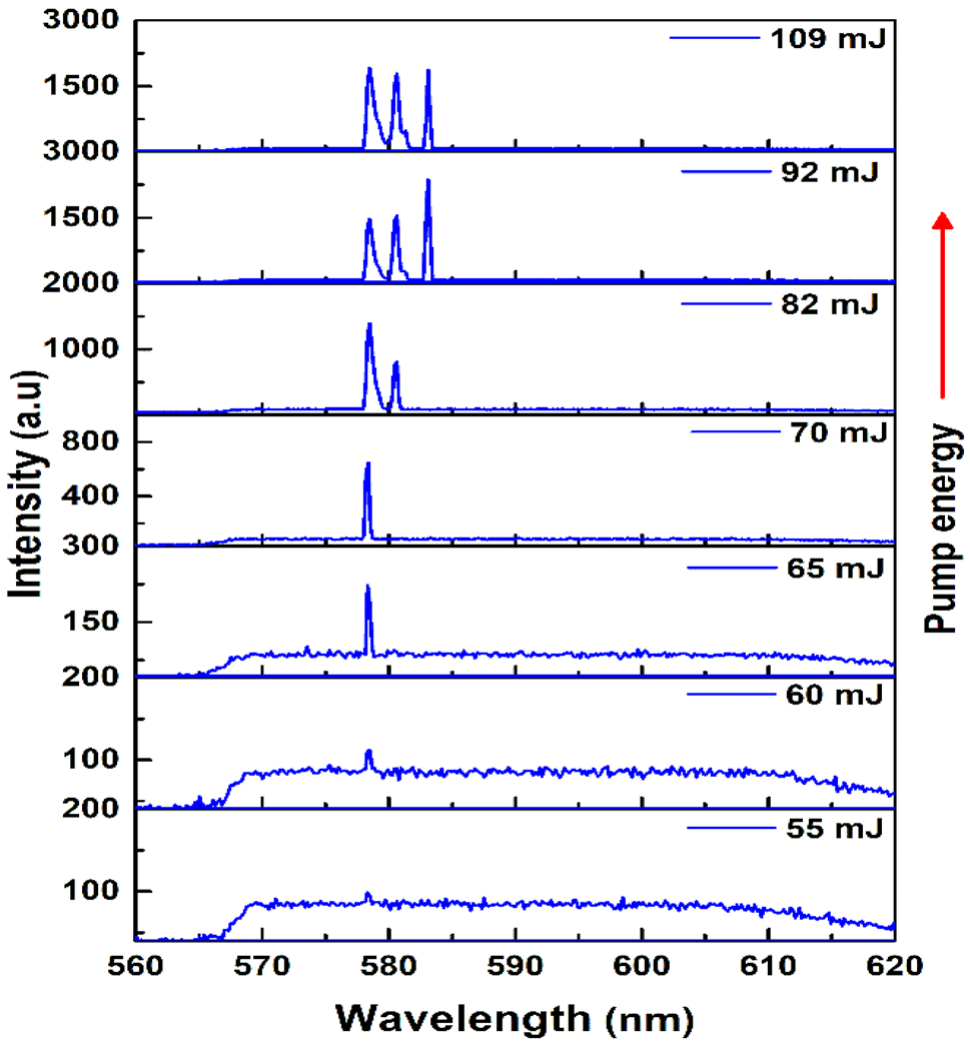
Evolution of the emission spectrum of Rhodamine 6G containing ZnO microspheres as a function of the pump energies.

Figure 3 shows the evolution of the emission spectra as a function of the excitation pump energy. At low pump pulse energy, spontaneous Raman scattering and fluorescence exhibit themselves as a broad spectrum. Once the pump energy reaches a certain threshold, the first Raman signal at a wavelength of 578 nm with a bandwidth of about 0.4 nm begins to grow. By gradually increasing the pump energy, the intensity of the single Raman signal increases, and the emission background decreases. The exponential growth of the Raman photons above the threshold due to the SRS process is responsible for this enhancement^37^. As the increase in the pump energy is ongoing, the second Raman signal appears at 580 nm wavelength. One can conclude that when the intensity of the first Raman signal becomes sufficient, this signal can act as a pump to produce the next Stokes by SRS. Without previous Raman signal, it is impossible to obtain a higher Raman signal in a cascaded SRS process^38^. As shown in Fig. 3, at higher pump energy, the third Raman signal manifests itself at the wavelength of 583 nm due to the generation of higher Raman signals at the higher pump energies^3^.

Observation of the cascaded SRS effect requires both high Raman gain of the medium and high pump power^6^. Using intense pump energy supports the required gain and is a key factor for the cascaded SRS effect occurrence. In addition, strong multiple elastic scattering increases the path length, and nonlinear interaction of photons results in the decreased threshold and favors Raman amplification^19^. Therefore, a proper selection of the scatterer centers to provide effective multiple elastic scattering is essential.

The increased surface area of microspheres due to porous surface structures provides high scattering efficiency. Furthermore, each nano-protrusion on the surface can act similar to a reflector for multiple elastic scattering. Consequently, efficient multiple elastic scattering requirements could be satisfied using the mentioned microspheres' specific morphology as scatterer centers.

**Figure 4:**
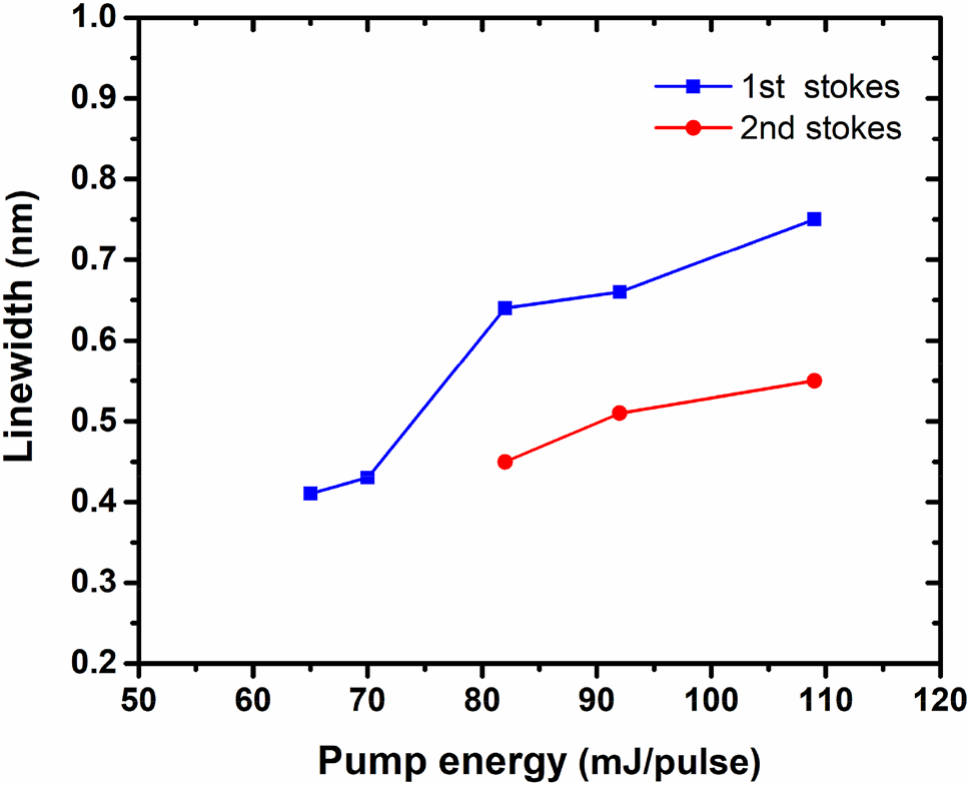
The linewidth (FWHM) of 1st Stokes (blue squares) and 2nd Raman Stokes (red circles) as a function of the input pump energy.

Figure 4 shows the variations of the linewidths versus the input pump energy for the first and second stokes signals. A slightly observed broadening in both Raman stokes with increasing the pump energy is attributed to the more intense nonlinear effects such as self phase modulation (SPM) at higher pump energies^4,5,27^.

**Figure 5:**
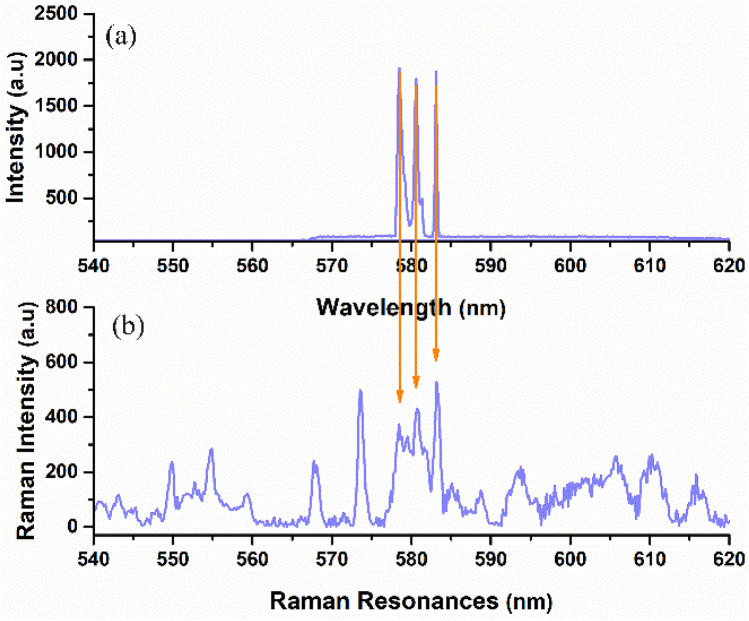
(**a**) Emission spectrum from Rhodamine 6G containing ZnO microspheres at 109 mJ/pulse pump energy. (**b**) The Raman spectrum of pure Rhodamine 6G dye. The arrows show the correspondence of the emission spectrum of Rhodamine 6G dye-containing ZnO microspheres and the Raman signals of pure Rhodamine 6G dye.

In Fig. 5, the spontaneous Raman spectrum of Rhodamine 6G was measured and compared with the emission spectrum of Rhodamine 6G containing ZnO microspheres. All three signals coincide with Raman signals of Rhodamine 6G dye, which provides evidence for the random Raman lasing process and confirms that the lasing modes originate from Rhodamine 6G Raman resonance.

**Figure 6:**
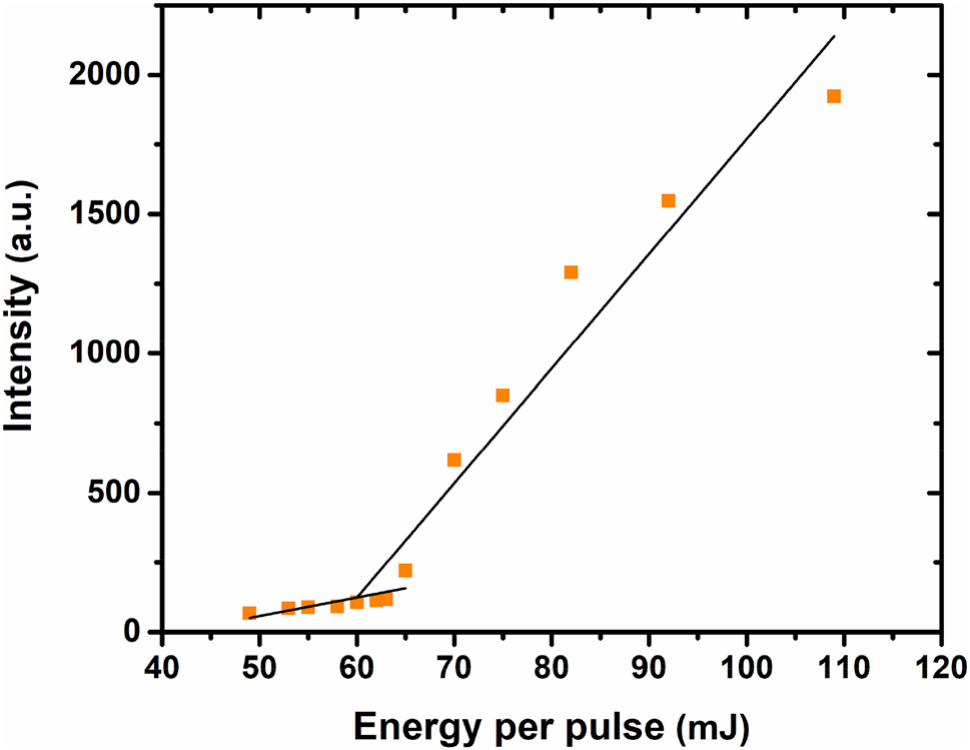
Output intensity versus incident pump energy at the first Raman signal (578 nm) from Rhodamine 6G solution containing ZnO microspheres.

Figure 6 shows the output Raman intensity of the first signal (578 nm) versus pump energy. A clear threshold for the Raman signal is observed to estimate the value for the transition from spontaneous scattering to SRS to be about 60 mJ/Pulse. The evolution of Raman signal intensity versus pump energy shows a nonlinear behavior. It is because the SRS intensity depends on both pump and random laser intensities^11,21^. Generally, output intensity in random lasers due to the absence of a stationary cavity is influenced by the multiple elastic scattering, which is occurred randomly at different directions^39,40^. Particularly, the fluctuations in the output intensity are further in solution random lasers because the Brownian motion of particles makes different configurations of scatterers at each excitation pulse^41^. On the other hand, in a dye solution, the reabsorption process due to the overlap of emission and absorbance spectrums of the dye is a factor of loss where many photons are absorbed before they leave the sample^42^.

**Figure 7:**
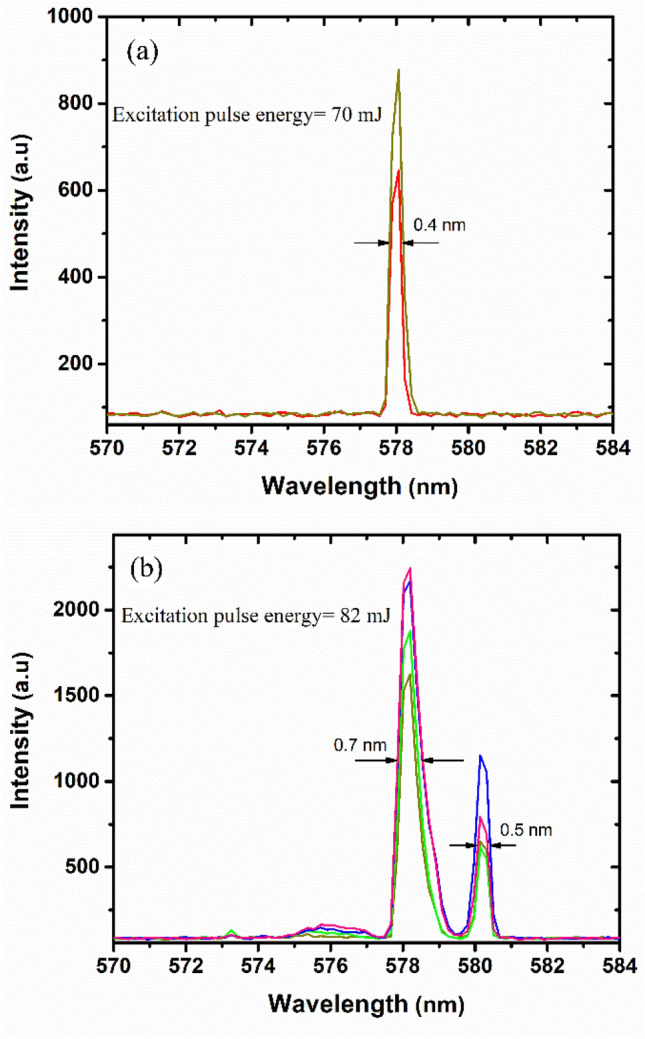
The behavior of Raman signals at independent single shots at (**a**) 70 mJ/pulse and (**b**) 82 mJ/pulse in Rhodamine 6G solution containing ZnO microspheres.

Figure 7a,b show the evolution of the Raman signals under multiple pulses at fixed energies of 70 and 82 mJ/pulse, respectively. As can see in Fig. 7a, at an excitation energy of 70 mJ/pulse, the Raman signal with a wavelength of 578 nm and a bandwidth of 0.4 nm reproduces well as the same as Fig. 3, for each excitation pulse. A similar result is observed at higher pump energy of 82 mJ/pulse, and Raman signals with wavelengths of 578 and 580 nm with relative linewidths of 0.7 and 0.5 nm are appeared, respectively. However, the intensity of Raman modes changes from pulse to pulse due to the dependence of SRS generation on scattering and absorption of photons^37^. Moreover, in a disordered solution medium, the distribution of scattering centers varied frequently due to the free movements of particles results in the partial variation of scattering intensity.

This experimental result reveals that the wavelengths of Raman signals are unchangeable from one pump pulse to another while only their emission intensity changes slightly.

## Conclusion

This study reports the first experimental observation of cascaded SRS effect in a colloidal Raman gain medium under intense pump energy.

Generation of cascaded SRS signals requires both a high gain and high pump power in the medium. Intense pump energy has been used to achieve a high gain and possibility for the transition from spontaneous Raman scattering to stimulated ones. Moreover, Raman amplification is increased by increasing the path lengths of photons inside the Raman gain medium via nano-protrusion on the surface of ZnO microspheres because of produce additional SRS processes.

The structure of ZnO microspheres plays an important role in providing strong multiple elastic scattering and consequently enhances the cascaded SRS generation. Furthermore, the coincidence of observed peaks on the spontaneous Raman signals of Rhodamine 6G provided experimental evidence for the presence of random Raman lasing and revealed the origin of lasing modes.

The evolution of output emission energy versus incident pump energy shows a threshold of about 60 mJ/pulse for the appearance of the first Raman signal. Furthermore, the evolution of Raman signals is considered under several fixed pump pulses. The results show that Raman signals are stable from one pulse to another. However, their intensities can change because SRS generation is dependent on the scattering and absorption of photons inside the medium.

Our findings offer a possibility to achieve the single-frequency laser sources (which can be achieved difficult and expensive) in a simple experimental setup with low-cost material using the cascaded SRS effect. Moreover, the cascaded random Raman laser is promising for tuning the wavelength of random lasers to generate desirable wavelengths for specific applications.

## Data Availability

The data supporting this study's findings are available from the corresponding author (E.Y) upon reasonable request.
